# Angle-Sensitive Photonic Crystals for Simultaneous Detection and Photocatalytic Degradation of Hazardous Diazo Compounds

**DOI:** 10.3390/mi11010093

**Published:** 2020-01-15

**Authors:** Kenichi Maeno, Bhargav R. Patel, Tatsuro Endo, Kagan Kerman

**Affiliations:** 1 Department of Applied Chemistry, Osaka Prefecture University, 1-1, Gakuencho, Naka-ku, Sakai, Osaka 599-8531, Japan; 2 Department of Physical and Environmental Sciences, University of Toronto at Scarborough, 1265 Military Trail, Toronto, ON M1C 1A4, Canada

**Keywords:** photonic crystals, Congo Red, Amido Black 10B, photocatalytic, reflectance spectroscopy, TiO_2_, environmental sensor

## Abstract

Congo Red (CR) and Amido Black 10B (AB-10B) are anionic diazo dyes, which are metabolized to produce a bioaccumulative and persistent carcinogen, benzidine. In this regard, an angle sensitive sensor composed of photonic crystal supported photocatalyst was fabricated for the simultaneous detection and photocatalytic degradation of diazo dyes from aqueous solutions. Reflectance spectroscopy was used in the detection of CR and AB-10B, which was based on the emergence of the incident angle dependent reflection peaks from the TiO_2_ coated two-dimensional photonic crystal (2D-PhC) surfaces and their subsequent quenching due to the presence of dye molecules whose absorbance peak intensity overlapped the reflection peak intensity of TiO_2_ at the respective angle. Interestingly, ultraviolet (UV) mediated photocatalytic degradation of CR and AB-10B was achieved using the same TiO_2_ coated 2D-PhC surfaces. 2D-PhC underneath the TiO_2_ layer was able to confine and localize the light on the TiO_2_ coated 2D-PhC surface, which enhanced the light absorption by dye molecules on the TiO_2_ surface and the photocatalytic efficiency in the degradation of CR and AB-10B. Finally, this proof-of-concept study demonstrated the fabrication of copolymer film based photonic crystal supported photocatalytic device, which can be used for developing miniaturized sensors competent in on-field detection and degradation of pollutants.

## 1. Introduction

Azo dyes constitute about 60–70% of the 800,000 tonnes of synthetic dyes produced world wide annually [1,2]. Past studies have shown that even at lower concentrations, azo dyes are genotoxic, mutagenic, and carcinogenic and pose a great risk to ecological and human health due to their inherent toxicity [3]. Moreover, upon entering the body, azo dyes are known to bind with proteins and cause allergic reactions by metabolizing into benzidine through liver enzymes [1,2]. In this study Congo Red (CR), disodium;4-amino-3-[[4-[4-[(1-amino-4-sulfonatonaphthalen-2-yl)diazenyl] phenyl]phenyl]diazenyl]naphthalene-1-sulfonate and Amido Black 10B (AB-10B), disodium;4-amino-5-hydroxy-3-[(4-nitrophenyl)diazenyl]-6-phenyldiazenylnaphthalene-2,7-disulfonate (Figure S1), were chosen as our model diazo compounds due to their commercial appeal and vast potential applications. Both CR and AB-10 are hazardous anionic diazo dyes found in aquifers and wastewater effluents of biochemical laboratories and textile, paper and pulp, plastic, and rubber industries [4]. Several sensitive and efficient analytical methods have become available for monitoring and detecting trace levels of dyes in aqueous solutions [5]; however, these methods require highly skilled personnel to operate the sophisticated instrumentation and are only available in large centralized laboratories, hindering their use as routine monitoring applications.

Therefore, to protect the aqueous ecosystem and human health from the hazardous effects of diazo dyes, portable and rapid detection of the contaminant is one of the desirable characteristics of a sensor. However, if the same sensor can be used for degradation of the contaminants in the wastewater effluents before their discharge into the environment, the versatility of the device can be improved. Recently, photonic crystals (PhCs) have emerged as a promising tool for chemical and biosensing applications and development of optical sensors [6,7,8]. PhCs are formed from periodic nanostructures, possessing a photonic band gap (PBG), of different periodic dielectric constants, which manipulates the transmission of light within a certain wavelength situated in PBG, based on Bragg’s diffraction, permitting sensitive chemical or biological detection in optical sensors [9]. Two-dimensional (2D) PhC based optical sensors can become promising pre-screening alternatives for simultaneously detecting and destroying azo dyes in remote sensing areas due to their potential for miniaturization and low cost production [10,11].

The present study is intended to offer a proof-of-concept for an angle sensitive multipurpose device, which can be used for angle dependent simultaneous detection and degradation of environmentally hazardous diazo compounds (CR and AB-10B). Several studies have been reported in literature for the degradation of azo dyes using TiO_2_ nanoparticles, immobilized TiO_2_ films, or TiO_2_ powder, but very few studies have used these for the detection of azo compounds [4,12]. In our previous work [13], we showed that the TiO_2_ coated 2D-PhC nanoprobe can be used for detection and degradation of a triarylmethane dye at a fixed angle of incident light. However, to the best of our knowledge, there are no studies demonstrating the use of TiO_2_ coated 2D-PhC surfaces for angle dependent simultaneous detection and degradation of azo dyes. Moreover, we showed that the fabricated TiO_2_ coated 2D-PhC surface produced angle dependent structural colors due to its interaction with different incident angles of light in the visible region, which gave rise to angle dependent reflection peak maxima and strong iridescence. The detection of CR and AB-10B was performed using a reflectance based TiO_2_ coated 2D-PhC sensor. In addition, the synergistic effect of 2D-PhC and TiO_2_ was studied for the UV mediated photocatalytic degradation of the dyes. Finally, the efficiency and kinetic analyses in photodegradation of the model diazo compounds were performed, and the optical characteristics of the fabricated TiO_2_ coated 2D-PhC were determined using reflectance spectroscopy and UV-Vis spectrophotometry, while the surface characterization was performed using cross-sectional transmission electron microscopy (XTEM), scanning electron microscopy (SEM), and X-ray photoelectron spectroscopy (XPS). The results showed that the TiO_2_ coated 2D-PhC could develop angle dependent structural colors with distinct reflection peaks after their interaction with incident light at different angles, and the catalyst on the TiO_2_ coated 2D-PhC surface can be re-used for repeated measurements while not requiring any post-treatment steps (e.g., filtration or centrifugation) to the TiO_2_ coated 2D-PhC surface.

## 2. Materials and Methods 

### 2.1. Fabrication of the TiO_2_ Coated 2D-PhC 

The TiO_2_ coated 2D-PhC devices were prepared as described in detail previously [6,14]. Briefly, a cyclo-olefin polymer (COP) film having a surface made up of nanopillar arrays was used to fabricate the TiO_2_ coated 2D-PhC surfaces by forming a thin layer of TiO_2_ (90 nm) on the COP film using liquid phase deposition (LPD). The initial structure of COP was sketched as a triangular lattice with a lattice constant of 430 nm, a radius of 115 nm, and a height of 200 nm. A plasma cleaner (CUTE-MP (MP/R), Femto Science, Yongin, Korea) (100 W, air 20 sccm, 0.5 Torr, 1 min) was used to activate the COP film. Following this, the film was rinsed in LPD solutions of pH 3 comprised of 0.15 M diammonium hexafluorotitanate ((NH_4_)_2_TiF_6_) and 0.45 boric acid for 90 min at 40 °C. TiO_2_ coated 2D-PhC was fabricated and cleaned with deionized water. Subsequently, the TiO_2_ coated 2D-PhC was mounted on a PVC black plate with optical adhesive (NOA-81). NOA-81 is a colorless UV resin.

### 2.2. Reflectance Spectroscopy for Detection of CR and AB-10B

**Figure 1:**
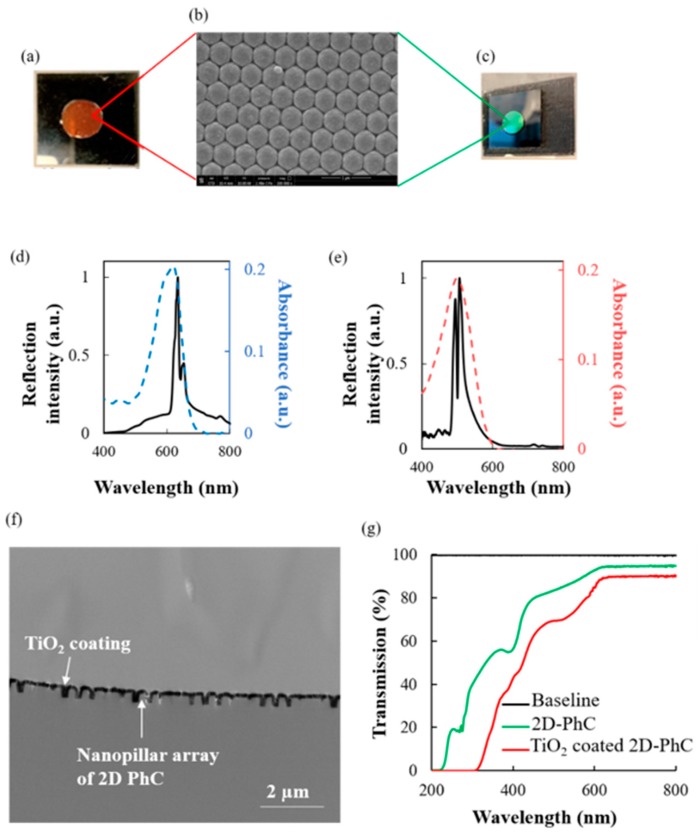
(**a**) Structural color observed from the TiO_2_ coated 2D- photonic crystal (PhC) surface when it is on a flat surface and the angle of incident light is 0°. (**b**) SEM image of the TiO_2_ coated 2D-PhC surface; the scale bar indicates 1 µm. (**c**) Structural color observed from the TiO_2_ coated 2D-PhC surface when it is on a flat surface and the angle of incident light is 60°. (**d**) Reflection spectra from TiO_2_ coated 2D-PhC when the incident angle for light is 0° (black solid line), absorption spectra of Amido Black 10B (AB-10B) (blue dashed line) and (**e**) Reflection spectra from TiO_2_ coated 2D-PhC when the incident angle for light is 60° (black solid line) and absorption spectra of Congo Red (CR) (red dashed line). (**f**) Cross-sectional profile done using XTEM, which shows the nanopillar array and the TiO_2_ coating on the 2D-PhC. (**g**) Transmission spectra of cycloolefin polymer based 2D-PhC and TiO_2_ coated 2D-PhC.

The detection of AB-10B (molar absorptivity (*ε*), 43,684 L·mol^−1^·cm^−1^), and CR (*ε*, 62,600 L·mol^−1^·cm^−1^) was performed using the TiO_2_ coated 2D-PhC as a sensing element that was aligned as shown in the optical setup in Figures S2 and S3, respectively, through reflectance spectroscopy [15,16]. At a 0° angle of incidence, the peak maxima for TiO_2_ coated 2D-PhC in dH_2_O was observed at a wavelength (*λ*) of ~630 nm and was used as a reference for the detection of AB-10B. In addition, the observed absorbance peak maxima of AB-10B at a wavelength of ~620 nm matched the reflection peak maxima at ~630 nm, and it was used in the trace detection of AB-10B. Similarly, at a 60° angle of incidence, the peak maxima for TiO_2_ coated 2D-PhC in dH_2_O was observed at a wavelength (*λ*) of ~494 nm and was used as a reference for the detection of CR. In addition, the observed absorbance peak maxima of CR at a wavelength of ~495 nm matched the reflection peak maxima at ~494 nm, and this was used in the trace detection of CR (Figure 1). Consequently, the reflection intensity of the TiO_2_ coated 2D-PhC decreased with an increase in the dye concentrations at both incident angles. Therefore, for the calibration plot and trace detection of AB-10B and CR, varying concentrations (1–20 µM) of the dyes were added onto the TiO_2_ coated 2D-PhC aligned a at 0° and a 60° angle of incidence, respectively, and the changes in the peak maxima at ~630 nm for AB-10B and ~494 nm for CR were recorded. The potential applicability of the angle dependent sensor was examined by detecting CR or AB-10B individually from a binary mixture containing a 1:1 ratio of both anionic diazo dyes in creek water (Highland Creek, ON, Canada). The recovery of CR and AB-10B was determined in the presence of interfering dye solutions using the aforementioned detection protocols at different incident angles in triplicate (*n* = 3). 

### 2.3. Photocatalytic Degradation of CR and AB-10B

The photocatalytic efficiency of the TiO_2_ coated 2D-PhC was analyzed by monitoring the photodegradation of CR and AB-10B under short UV irradiation at 254 nm (8.5 µW/cm^2^) using a UVGL-15 compact UV lamp (Upland, CA, USA) as the radiation source at a distance of 0.1 cm. An aliquot (~200 µL) of CR and AB-10B dye solution was placed in the reaction chamber made using a PDMS well on the top of TiO_2_ coated 2D-PhC (diameter: 8 mm), and the photocatalytic process was performed. Moreover, the 2D-PhC without TiO_2_ and TiO_2_ nano-powder immobilised on the glass surface were used as a control to depict the synergistic effect of TiO_2_ and 2D-PhC. The initial CR and AB-10B dye concentration was optimized to be at 20 µM, since it was significant in assisting the visual readouts after the photodegradation at different timepoints. At regular timepoints after the UV irradiation, the CR and AB-10B samples were transferred from the PDMS well into the 96 well microplate. Following this, UV analysis was performed using a Biotek Synergy^®^ H1 microplate reader (Winooski, VT, USA), to monitor the photocatalytic degradation from the spectral changes of the respective dyes before and after the exposure to UV irradiation. An aliquot of the CR and AB-10B was placed in a microplate, and a UV-Vis scan from 320 nm to 800 nm at a resolution of 5 nm was performed. The absorbance values at ~494 for CR and ~630 nm for AB-10B (*λ* max) were measured before and after UV irradiation. The degradation rate (*η*) of the respective dyes on TiO_2_ coated 2D-PhC was calculated by Equation (1) [17]:

*η* = 1 − (*A*_t_/*A*_o_) × 100 (1)

Here, *A*_0_ and *A*_t_, are the absorption intensities of CR and AB-10B dye before and after the photocatalytic reaction at a certain time interval, respectively.

## 3. Results

### 3.1. Production of TiO_2_ Coated 2D-PhC

**Figure 2:**
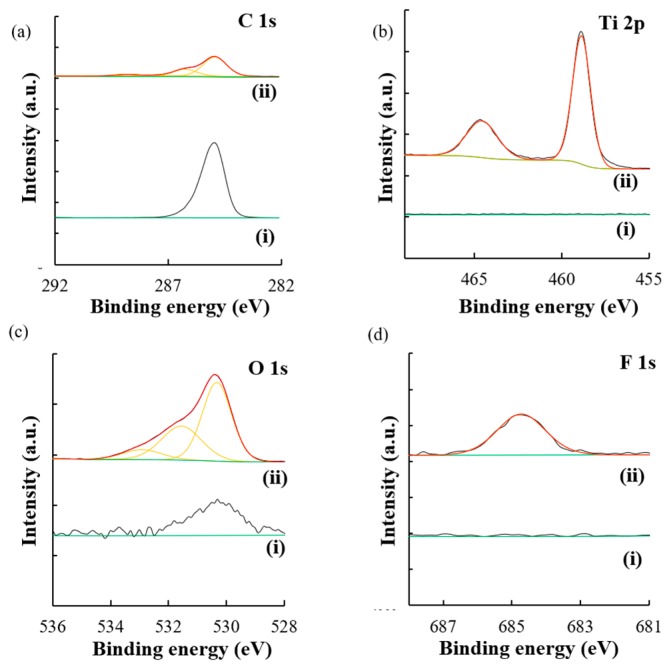
Photoelectron analyses of 2D-PhC surfaces (i) and TiO_2_ coated 2D-PhC surfaces (ii): (**a**) C 1s spectra, (**b**) Ti 2p spectra, (**c**) O 1s spectra, and (**d**) F 1s spectra. Experimental details of XPS survey spectra are defined in the Electronic Supplementary Materials (ESM) and shown in Figure S4.

LPD was the chosen technique for the fabrication of TiO_2_ coated 2D-PhCs, which resulted in a large surface area with optical properties in the visible region. Furthermore, molecular information and confirmation of the TiO_2_ coated 2D-PhC were obtained with X-ray photoelectric spectroscopy (XPS). In Figure 2a–d, (i) is the signal obtained for the specific element on the bare 2D-PhC without any TiO_2_ coating, while (ii) is the signal obtained for the specific element after the TiO_2_ coating on the 2D-PhC. After the coating of the TiO_2_ on the 2D-PhC (ii), the Ti 2p spectra at 458.8 and 464.5 eV and the O 1s spectrum shown at 530.3 eV correlated with the TiO_2_ coating on the 2D-PhC. The F 1s spectrum shown at 684.6 eV also indicated that the TiO_2_ was liquid phase deposited on the 2D-PhC from the (NH_4_)_2_TiF_6_ used during fabrication. In the C 1s spectrum, a high intensity of the carbon signal was observed prior to modification (Figure 2ai). After the surface modification process (Figure 2aii), a decrease in the intensity of the observed signal of carbon in the C 1s spectrum at 284.95 eV was observed, which indicated some modification on the carbon surface. Finally, for the O 1s spectrum, compared to the uncoated surface (Figure 2ci), the TiO_2_ coated 2D-PhC surface showed an increase of the signal at the 530.3 eV position in Figure 2cii, which indicated that some new oxygen species were present.

**Figure 3:**
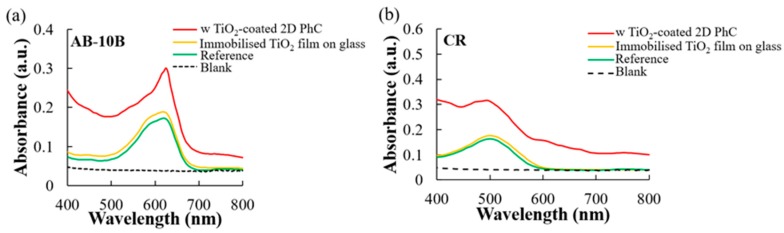
Enhancement in the absorption intensity of peak maxima for (**a**) AB-10B and (**b**) CR with TiO_2_ coated 2D-PhC compared to peak maxima in the presence of TiO_2_ immobilized as a film on glass and the peak maxima of the dye solutions in absence of both TiO_2_ coating in any form and 2D-PhC.

In this study, the TiO_2_ coated 2D-PhC surface with incident angle dependent structural colors capable of sensing different diazo dyes was developed. At a 0° angle of incidence, the sensor showed two reflection peaks at ~630 nm and ~655 nm in dH_2_O, as illustrated in Figure 1d. Similarly, at a 60° angle of incidence of white light, the device showed two reflection peaks at ~494 nm and ~505 nm in dH_2_O under the Scope mode of the fiber optic bundle, as illustrated in Figure 1e. These peaks were designed to overlap with the absorption peak maxima (~495 nm and ~620 nm) of the target diazo compounds, CR and AB-10B, and were also predicted to be derived from resonant modes caused by stop bands of TiO_2_ coated 2D-PhC when the incident angle for light was 60° and 0°, respectively. Finally, due to the presence of 2D-PhC, it was shown (Figure 3) that the confined light was localized on the TiO_2_ coated 2D-PhC surface, and the absorption intensity of peak maxima for the target dyes, CR and AB-10B, on the surface of the constructed device was enhanced by 73.37 ± 2.46% for CR and 67.82 ± 1.45% for AB-10B, compared to the reference [18]. Therefore, as shown in Figure 3, both 2D-PhC and the TiO_2_ coating were necessary for causing the absorption enhancement for the dyes. Furthermore, in the absence of the 2D-PhC, there was no obvious enhancement in the absorption of the peak maxima even in the presence of immobilized TiO_2_ film on glass surface, when compared to the reference (in absence of both TiO_2_ coating and 2D-PhC).

### 3.2. Detection of CR and AB-10B

**Figure 4:**
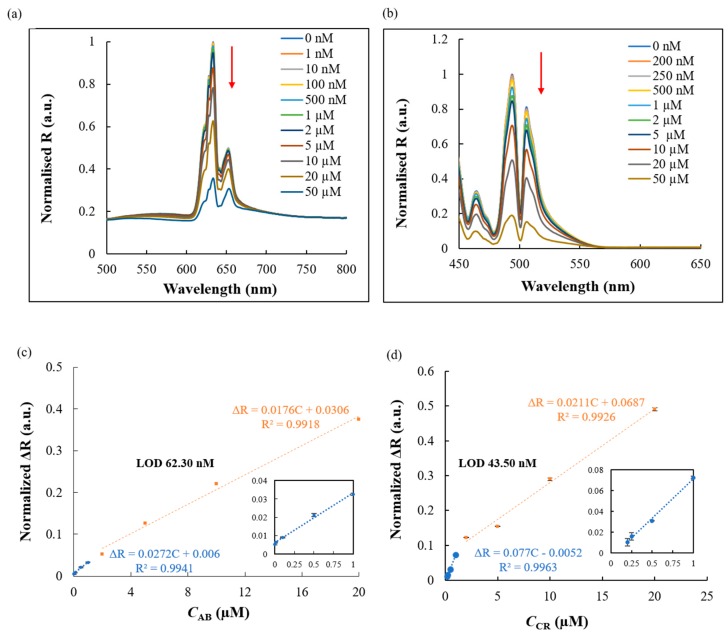
Angle dependent detection of diazo dyes using TiO_2_ coated 2D-PhC. Reflection spectrum of TiO_2_ coated 2D-PhC corresponding to the different concentrations of (**a**) AB-10B at a 0° angle of incidence and (**b**) CR at a 60° angle of incidence. Calibration curve for the detection of (**c**) AB-10B and (**d**) CR from 0–20 µM. The inset represents the linear relationship observed at lower concentrations for AB-10B (0.001–1 µM) and CR (0.2–1 µM).

In the present study, the fabricated TiO_2_ coated 2D-PhC surface resulted in the production of incident angle dependent iridescent structural colors with a reflection spectrum having peak maxima at a wavelength in the visible region (Figure 1). Herein, the usability of this device was evaluated by performing simultaneous detection of diazo dyes, CR and AB-10B, in aqueous solutions and from a binary mixture of both dyes. In the presence of perpendicularly irradiated white light at incident angles of 0° and 60°, various concentrations of AB-10B and CR were applied on the TiO_2_ coated 2D-PhC surface, respectively, and the corresponding reflection spectra were monitored. As shown in Figure 4a,b, the reflection intensities of the TiO_2_ coated 2D-PhC gradually decreased with increasing AB-10B and CR concentrations. We hypothesized that the simultaneous absorption enhancement of CR and AB-10B observed at the respective incident angles, due to the TiO_2_ coated 2D-PhC, caused this decrease in the reflection spectra. An approximately six fold absorption enhancement for larger nanostructures of TiO_2_ surface was reported in the literature [19]. Finally, past research has also attributed the absorption enhancement to the generation of titanium rich zones and crystalline TiO_2_ nanostructures, specifically after the LPD of TiO_2_ on the 2D-PhC surfaces, as reported in this study [18,19].

The variations in the reflection intensity were shown from 1 nM to 50 µM for AB-10B and CR. The plot for the normalised reflection intensity of AB-10B and CR at the surface of TiO_2_ coated 2D-PhC was linearly dependent on the increasing dye concentrations in two linear segments with two different slopes associated with two different ranges of dye concentration (Figure 4c,d). Upon monitoring the optical characteristics of TiO_2_ coated 2D-PhC at a 0° and a 60° angle of incidence, respectively, the reflection spectrum was normalised using the following equation: Δ*R* = 1 − *R*_Normalised_, where *R*_Normalised_ = (*R*_c_/*R*_Reference_)/*R*_max,0_, wherein *R*_c_ is the reflection intensity of the target dye at different concentrations, *R*_Reference_ is the background reflection intensity measured using the Lambertian reference surface comprised of a WS-1 diffuse reflectance standard, and *R*_max,0_ is the maximum reflection intensity measured in the absence of any dye. We also hypothesized that at low and high concentrations of the diazo dyes, the surface activity of the angle sensitive TiO_2_ coated 2D-PhC would be different, which was responsible for the observed difference in the slopes of two linear segments. A high number of active sites was available on the TiO_2_ coated 2D-PhC surface at lower concentrations of the model dyes. However, the number of active sites decreased at higher concentrations of the model dyes. The different number of active sites resulted in the decreased sensitivity of the slope in the second linear segments for AB-10B and CR, respectively. The relationship of the limit of detection (LOD) = 3 × SD_blank_/b [20] was used to derive the experimental detection limit for AB-10B (62.30 nM) and CR (43.50 nM), where SD_blank_ is the relative standard deviation of blank signals (*n* = 3) and b is the slope of the calibration plot. 

Moreover, the wavelength of reflection peak maxima from the fabricated TiO_2_ coated 2D-PhC was tuned and blue shifted with increasing angle of incidence, in conjunction with the structural color of the device that was angle dependent. When the angle of the TiO_2_ coated 2D-PhC changed from 0° to 60°, the colors of the TiO_2_ coated 2D-PhC changed from red to green (Figure 1a,c, respectively), and the positions of the major reflection peaks varied from ~630 nm to ~494 nm (Figure 1d,e). This shift in the wavelength of maximum reflection peak was consistent with Bragg’s law and has been reported as a typical feature for photonic crystals in past research [21,22]. Based on the past studies using 2D-PhC based sensors [9,14,23,24], the relationship between the angle of incidence, the angle sensitive iridescent structural color, and the Bragg peaks observed from the TiO_2_ coated 2D-PhC were used to detect the target dye molecules using Bragg’s law as follows [23,25]:

*mλ* = 2*nd*sin*θ* (2)

where *m* is the diffraction order, *λ* is the wavelength of the Bragg peak (reflected light) at a specific angle of incidence, n is the average refractive index (RI), d is the distance of the diffracting plane spacing for 2D materials in the lattice, and *θ* is the incident angle for light exposed onto the TiO_2_ coated 2D-PhC. Here, it should be noted that *θ* is defined relative to the surface of the reflecting plane rather than a normal to that plane. Therefore, the position of the Bragg peak and the structural colors can be tuned by the incident angle and the manipulation of refractive indices of polymer materials used. Hence, the peak wavelength was used for simultaneous detection of diazo dyes from binary mixtures at different angles of incidence. Specifically, in the present study, the RI change caused by the TiO_2_ and the dye solutions’ interaction was monitored by the quenching in the reflection peak intensity that was based on Fresnel reflection [23,24]. The behaviour of the light when it moves between media having different RIs results in the Fresnel reflection. When the Fresnel reflection for the incident light is straight compared to the interface of two media, the parallel and perpendicular components of the light will not have any significant difference between them. Furthermore, control assays for the detection of diazo dyes individually in aqueous solutions without 2D-PhC and TiO_2_ were also carried out; however, there was no observable change in the reflection intensity response at different concentrations.

**Figure 5:**
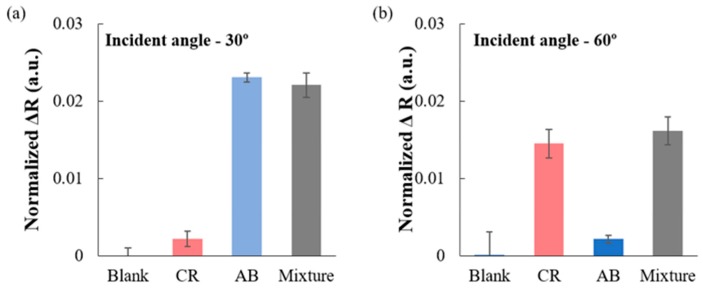
(**a**) Recovery of AB-10B (500 nM) using the TiO_2_ coated 2D-PhC from the 1:1 (500 nM) binary mixture of AB-10B and CR when the light was irradiated at an incident angle of 0°. (**b**) Recovery of CR (500 nM) using the TiO_2_ coated 2D-PhC from the 1:1 (500 nM) binary mixture of AB-10B and CR when the light was irradiated at an incident angle of 60°.

After the calibration studies, the practicality of TiO_2_ coated 2D-PhC was tested for incident angle dependent detection of CR and AB-10B from the binary mixture of both dyes prepared in creek water (Highland Creek, ON, Canada) using the spike and recovery method. The results (Figure 5a) showed that when the angle of incidence for the light was 0°, AB-10B was successfully detected, and when the angle of incidence for the light was changed to 60°, CR (Figure 5b) was successfully detected from the 1:1 binary mixture of anionic dyes, CR and AB-10B. 

Therefore, in the binary mixtures, each dye at the corresponding Bragg angle of incidence produced a significant change in the reflection intensity spectra of TiO_2_ coated 2D-PhC surfaces and, therefore, was detected with good recovery in a complex matrix. Finally, the TiO_2_ coated 2D-PhC did not show a significant change in the reflection intensity and the wavelength of the peak maxima for CR and AB-10B for more than one month; such results proved the stability of the TiO_2_ coated 2D-PhC device. Additionally, the performance of the angle sensitive sensor was compared with other techniques used to detect azo dyes in the literature (Table 1). 

It was observed that our proposed sensor had a wide linear range and low limit of detection compared to the other techniques. Moreover, unlike the majority of the other sensors and techniques, the proposed sensor could be used for as a multipurpose device for the detection and degradation of azo dyes.

### 3.3. Photocatalytic Degradation of CR and AB-10B

Following the photocatalytic degradation of CR and AB-10B as described earlier, the UV-visible absorption spectra for both diazo dyes were measured before and after the UV exposure at different time points (Figure S5a,b). It was hypothesized that owing to the fragmentation of TiO_2_ located on the 2D-PhC nanostructures, the dye molecules had accessibility to a greater surface area on TiO_2_ coated 2D-PhC, which led to an increased decolorization and photodegradation in the presence of the TiO_2_ coated 2D-PhC surface [32]. 

**Figure 6:**
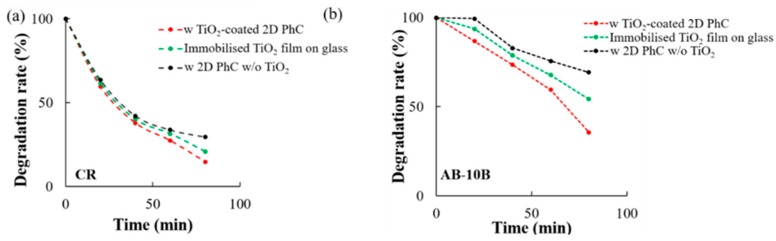
UV mediated photocatalytic degradation efficiency of TiO_2_ coated 2D-PhC. UV mediated photodegradation of 20 µM (**a**) CR and (**b**) AB-10B with TiO_2_ coated 2D-PhC, TiO_2_ nano-powder immobilized on the glass surface, and 2D-PhC without TiO_2_ at different time points.

In addition, using Equation (1), the UV mediated photocatalytic activity and the photodegradation efficiency of the TiO_2_ coated 2D-PhC were investigated (Figure 6 and Figure S5). The results demonstrated that the degradation rate (%) of CR and AB-10B was significantly increased when the experiment was performed in the presence of the TiO_2_ coated 2D-PhC surface compared to the photodegradation performed using only the 2D-PhC COP film (i.e., without TiO_2_) and the TiO_2_ nano-powder immobilized glass surface (Figure 6).

**Figure 7:**
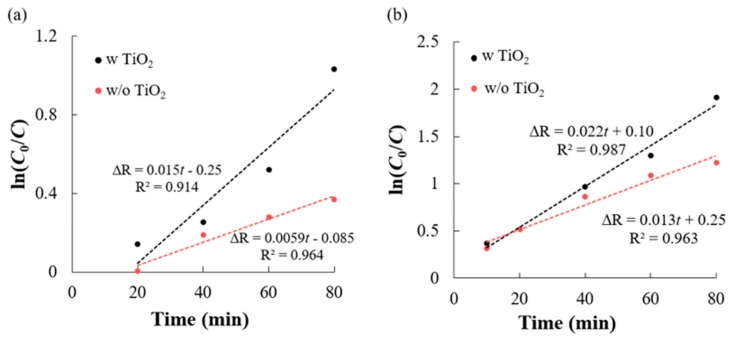
Langmuir–Hinshelwood plots showing pseudo-first order decolorization reaction kinetics for 20 µM of (**a**) AB-10B and (**b**) CR with and without TiO_2_ coated 2D-PhC under UV irradiation at different time points.

The Langmuir–Hinshelwood (L-H) kinetics model was used to quantify the rate of photodegradation reaction occurring on the surface of the photocatalyst (TiO_2_) under UV irradiation [33]. The different photocatalytic degradation rates of CR and AB-10B in the presence and absence of TiO_2_ coated 2D-PhC were identified to follow a pseudo-first order kinetics (Figure 7) using the equations shown in Electronic Supplementary Materials (ESM). In the presence of TiO_2_ coated 2D-PhC, the photodegradation rate constants for CR and AB-10B were 0.022/min and 0.015/min, respectively, while in its absence, the rates for CR and AB-10B were 0.013/min and 0.0059/min, respectively. 

Moreover, as shown in Figure S6, the TiO_2_ coated 2D-PhC was used for 20 successive trials to perform photocatalytic degradation under UV irradiation. The photocatalytic degradation rate (%) at 20 min was compared for the 20 successive trials, and the results shown in Figure S6 emphasized that the relative standard deviations of the results were 9.54 and 3.95% for AB-10B and CR, respectively. These results confirmed the excellent repeatability for the TiO_2_ coated 2D-PhC in the degradation of the target dyes. Furthermore, the stability and reusability of the TiO_2_ coated 2D-PhC were investigated by using it to measure reflection intensity signals for the detection of 1 µM AB-10B and CR before and after 20 successive UV mediated photocatalytic degradation cycles. The stability of the TiO_2_ coated 2D-PhC was emphasized by the retained reflection peak maxima (normalised R) values for AB-10B and CR at 95.36% and 97.44%, respectively.

Furthermore, as established in this study, the photon lifetime could be elongated on the PhC surface, and the optical absorption could be enhanced because the PhCs had unique optical characteristics that allowed them to manipulate and confine the light incident on their surface [34]. In addition, the nanostructured materials such as PhC supported photocatalysts were reported to show an increase in their photocatalytic degradation efficiency [17,35]. Therefore, it was shown that the 2D-PhC underneath the TiO_2_ coating played a synergistic role in enhancing the rate of UV mediated photocatalytic degradation of CR and AB-10 significantly after the timepoints of 60 and 80 min. The highly ordered and interconnected channels of the 2D-PhC nanostructures increased the electron charge transfer onto the TiO_2_ active sites, which was advantageous in aiding the transfer of dye molecules to the surface of TiO_2_, promoting the enhancement in the overall photocatalytic activity [36]. 

## 4. Conclusions

The practicality of the angle sensitive TiO_2_ coated 2D-PhC surfaces, for incident angle dependent simultaneous detection and degradation of toxic diazo dyes, CR and AB-10B, was examined. The detection of CR and AB-10B individually and from the binary interfering dye mixtures was highly reproducible, rapid (~45 s), and cost effective. Our study demonstrated that TiO_2_ coated 2D-PhCs can be a promising pre-screening tool for rapid and simultaneous detection of different dyes from a mixture by just tuning the incident angle of light projected on the TiO_2_ coated 2D-PhC surface. Moreover, TiO_2_ coated 2D-PhCs are also a promising platform in remediation and wastewater treatment because of their capability of efficient photocatalytic degradation of diazo dyes. The solid state TiO_2_ coated 2D-PhC devices attained a high surface area from the photocatalyst and the 2D-PhC, making them stable and allowing them to be re-used for consecutive measurements, while not requiring any post-treatment steps (e.g., separation, filtration, or centrifugation) after the degradation processes, thereby solving the problems of photocatalyst sustainability and the hazardous impact of nanomaterials in the treated water. The potential application of lightweight, flexible, and angle sensitive TiO_2_ coated 2D-PhC devices in a commercialized wastewater treatment facility was emphasized through this study. 

## Figures and Tables

**Table 1:** Performance comparison and figure of merit for the proposed TiO_2_ coated 2D-PhC sensor with other detection methods.

Sensor Material	Detection	Degradation	Analytical Method	Linear Range (µM)	LOD (µM)	Ref.
**MIP@β-CD-MA**	Yes	No	SPE	0.0001–0.006	0.0001	[26]
**Bismuth film-GCE**	Yes	No	Electrochemistry	0–119	5.97	[27]
**Bismuth film-GCE**	Yes	No	Electrochemistry	0–83	1.65	[27]
**Silver hydrosol**	Yes	No	SERS	0–0.03	0.00002	[28]
**CElect-FS75 CE column**	Yes	No	Capillary zone electrophoresis	0.5–28	9.43	[29]
**Fe_3_O_4_ activated carbon-fullerene adsorbent**	Yes	No	MSPE-CE	11–75	3.74	[30]
**Graphene oxide-GCE**	Yes	Yes	Electrochemistry	0.01–0.2	0.24	[31]
**TiO_2_ coated 2D-PhC**	Yes	Yes	Reflection Spectroscopy	CR: 0.2–1 and 1–20 AB-10B: 0.001–1 and 1–20	CR: 0.0435 AB-10B: 0.0623	This work

MIP@β-CD-MA: molecularly imprinted layer-beta-cyclodextrins-maleic anhydride solid phase extraction (SPE), GCE: glassy carbon electrode, SERS: surface enhanced Raman spectroscopy, Fe_3_O_4_: magnetite, MSPE-CE: magnetic solid phase extraction-capillary electrophoresis.

## Supplementary Materials

The following are available online at https://www.mdpi.com/2072-666X/11/1/93/s1. A. Supplementary Methods, B. Mechanism of UV assisted photocatalytic degradation of MG on TiO_2_ coated 2D-PhC, C. Langmuir–Hinshelwood kinetic model, Figure S1: Molecular structure of model dyes, Figure S2: Schematic of the experimental setup for detection of AB-10B, Figure S3: Schematic of the experimental setup for the detection of CR, Figure S4: XPS survey scans, Figure S5: UV-Vis absorbance results corresponding to the color change upon different UV irradiation times, Figure S6: Reusability tests for TiO_2_ coated 2D-PhC in the photocatalytic degradation of CR and AB-10B.
