# Supplementary material for: Angle-Sensitive Photonic Crystals for Simultaneous Detection and Photocatalytic Degradation of Hazardous Diazo Compounds
Source: Micromachines (Basel). 2020 Jan 15;11(1):93. doi: 10.3390/mi11010093 (PMC7020205; doi:10.3390/mi11010093)
Supplement: Supplementary file 1 [file micromachines-11-00093-s001.pdf]

micromachines

Supplementary Materials: Angle-Sensitive Photonic Crystals for Simultaneous Detection and Photocatalytic Degradation of Hazardous Diazo Compounds

Kenichi Maeno, Bhargav R. Patel, Tatsuro Endo and Kagan Kerman

A. Supplementary Methods

Materials

Diammonium hexafluorotitanate (IV) ($(NH_4)_2TiF_6$), boric acid, and hydrochloric acid (HCl) were acquired from Wako Pure Chemicals (Osaka, Japan). Cyclo-olefin polymer (COP) nanostructured film was provided by SCIVAX Corp. (Kanagawa, Japan). Optical adhesive (NOA-81) was purchased from Norland Products Inc. (Cranbury, USA). The polyvinyl chloride (PVC) black plate was obtained from Terasaka Co. Ltd. (Kyoto, Japan). Malachite green (MG) oxalate was procured from BioBasic (Markham, ON). The polydimethylsiloxane (PDMS) well was made by manually mixing and heat curing a 10 (base):1 (curing agent) ratio of SYLGARD™ 184 silicone elastomer base and curing agent, purchased from Dow Corning Corporation (Midland, MI, USA). Congo Red (CR) [1-naphthalenesulfonic acid, 3,3′-(4,4′-biphenylenebis (azo)) bis(4-amino-) disodium salt] and Amido Black 10B (AB-10B) [2,7-naphthalenedisulfonic acid, 4-amino-5-hydroxy-3-[(4-nitrophenyl) azo]-6-(phenylazo-) disodium salt] dyes were purchased from Sigma Aldrich (Oakville, ON). All the other reagents and chemicals were of analytical grade, and 18.2 MΩ cm ultra-pure Milli-Q water (dH_2O) (Millipore, Darmstadt, Germany) was used to prepare the dye solutions for each measurement.

Optical characterization and measurements: An optical setup composed of a tungsten halogen light source (LS-1, wavelength range: 360–2000 nm), an optical fiber probe bundle (R-200-7 UV/VIS, fiber core diameter: 200 m, wavelength range: 250–800 nm), and a hand-held spectrophotometer (USB-4000-UV-VIS, wavelength range: 200–1100 nm, Ocean Optics, Dunedin, USA) was used for the detection of CR and AB-10B, respectively, by monitoring the reflection spectra from TiO_2 coated 2D-PhC surfaces as reported previously [1–3]. A UV-Vis spectrophotometer was used to measure the reflection spectra between 400 and 800 nm in the Scope mode of SpectraSuite® (Dunedin, FL, USA) at room temperature to evaluate the spectral characteristics of the fabricated device. Briefly, white light (wavelength range: 400–800 nm) was perpendicularly irradiated at different angles of incidence (0° and 60° angles of incidence) onto the TiO_2 coated 2D-PhC thorough the optical fiber bundle. The reflected light from each angle of incidence was coupled with the detection fiber probe situated in the fiber optic probe, and it was analyzed using the spectrophotometer. The Lambertian reference surface was comprised of a WS-1 diffuse reflectance standard from Ocean Optics (Dunedin, USA). A fixed probe height (~1 mm above the sample surface) was maintained throughout the study for both 0° and 60° angles of incidence.

Scanning electron microscopy: SEM analysis of the TiO_2 coated 2D-PhC was performed using a Quanta FEG 250 ESEM™ scanning electron microscope from FEI Company (Minato, Japan). The TiO_2 coated 2D-PhC surfaces were sputtered by osmium using the SEM coating unit OPC60 osmium sputter coater from Filgen Inc. (Nagoya, Japan) at 1 mA plasma current for few seconds to obtain a 10 nm coating of osmium.

X-ray photoelectron spectroscopy: The ThermoFisher Scientific K-Alpha spectrometer (ThermoFisher Scientific—E. Grinstead, U.K.) was used to perform all the measurements. Low resolution survey spectra (nominal 900 μm spot, 100 eV pass energy), followed by high resolution spectra (nominal 900 μm spot, 100 eV pass energy) for all of the elemental regions observed on the TiO_2 coated 2D-PhC surfaces were collected. The system's combined e^-/Ar^+ flood gun was used to apply the charge compensation. The energy scale was adjusted to place the main C 1s peak at 284.98 eV for the 2D-PhC without TiO_2 coating and to the main O 1s at 530.42 eV for the TiO_2 coated

2D-PhC. All data processing was performed using the software supplied with the system (AVANTAGE 5.926).

B. Mechanism of UV Assisted Photocatalytic Degradation of MG on TiO_2 Coated 2D-PhC

The fabricated TiO_2 coated 2D-PhC was then used for the heterogenous photocatalytic degradation of diazo dyes in the presence of UV irradiation. The heterogeneous photocatalytic reaction involving photodegradation of dye was initiated by the UV mediated photoexcitation of a semiconductor such as TiO_2. The detailed mechanism of UV assisted photocatalytic degradation of dye on the TiO_2 coated 2D-PhC surface is discussed below and was also illustrated in detail previously [4,5].

Titanium dioxide (TiO_2) can be activated by the UV irradiation, and it has been commonly employed for photocatalytic degradation of organic pollutants such as dyes because it is a biologically and chemically inert, non-toxic, cost effective, and anti-corrosive material. Furthermore, it can efficiently catalyze the redox reactions due to its rapid electron transfer capabilities, and it has a preferable band gap energy and is photochemically stable [5–7]. The most commonly used forms of TiO_2 are anatase and rutile, which are known to have a large band gap energy of 3.2 and 3.02 eV, respectively. The TiO_2 is activated upon UV irradiation ($\lambda \leq 387$ nm) after absorption of photons that are either equal or greater than its band gap width and energy. This absorption eventually results in a charge separation and generation of excited high energy electron/hole pairs, which are strong reducing and oxidizing agents, respectively. Upon photoexcitation, the electrons are promoted from the valence band (h^+_{VB}) of the TiO_2 to the conduction band (e^-_{CB}) and diffuse onto the surface of the TiO_2, leaving behind an electron valency and positive hole in the valence band. The photogenerated positive holes (h^+) at the TiO_2 surface can oxidize the electron donors such as a water molecule or hydroxyl ion into the hydroxyl radical ($OH^{\bullet}$) or oxidize the organic dye pollutant to form $dye^{\bullet+}$, because they have a high oxidation potential. On the other hand, the photogenerated electrons in the conduction band can reduce the organic dye pollutants or reduce the electron acceptors such as oxygen, present in the dissolved water or adsorbed on the Ti surface, into the anion radical superoxide ($O^{\bullet-}$), which can also react with H^+ ions and produce hydroxyl radicals. Due to its powerful oxidizing properties, the majority of the azo dyes undergo degradation and are mineralized to non-toxic end-products [5]. When the photocatalyst (TiO_2) is supported by the photonic crystals (2D-PhC film), the 2D-PhC film can trap the photons on the TiO_2 surface upon photoexcitation; in addition, the reflection of PhC and the absorption of light on the TiO_2 are divided into distinct materials, which leads to a substantial increase in the photocatalytic activity [8,9]. The 2D-PhC below the TiO_2 can also assist in the localization of the incident UV light during the photoexcitation and cause enhancement in the optical absorption, which leads to the photogeneration of energetic electron-hole pairs and improved photon lifetime [9,10].

C. Langmuir–Hinshelwood Kinetic Model

The difference in the photodegradation rates was evaluated and quantified using the pseudo-first order Langmuir–Hinshelwood (L-H) kinetic model expressed through the equations below [4,7,11,12]:

$$r = -\frac{dC}{dt} = kC \quad (3)$$

where r is the oxidation rate of AB-10B or CR (mg/L·min), C is the concentration of CR and AB-10B in aqueous solution (mg/L), t is the UV exposure time (min), and k is the pseudo-first order photocatalytic reaction rate constant (min^{-1}).

Consequently, integrating Equation (3) with regards to time and concentration gives:

$$C_t = C_0 e^{(-kt)} \quad (4)$$

where C_t is the concentration of AB-10B and CR in aqueous solution (mg/L) at UV exposure time t (min) and C_0 is the initial concentration of AB-10B and CR (mg/L).

Finally, the pseudo-first order kinetic equation can be obtained by linearly transforming Equation (4), which is expressed as:

$$\ln\left(\frac{C_t}{C_0}\right) = -kt \quad (5)$$

The photodegradation efficiencies of the photocatalyst can be maximized as this model aids in recognizing key information associated with the mechanism of photocatalytic degradation and elimination of pollutants [12]. As shown in Figure 5a,b, after plotting and linearly fitting the graph of ln (C_t/C_0) as a function of UV exposure time (t), the pseudo-first order reaction rate constants (k) in the presence and absence of TiO_2 coated 2D-PhC were calculated from the slope of the line for both AB-10B and CR, respectively.

The increase in the rate of photocatalytic reaction by a factor of ~1.7 times for CR and ~2.5 times for AB-10B in the presence of TiO_2 coated 2D-PhC emphasized that TiO_2 and 2D-PhC were involved in the accelerated degradation of the respective diazo dyes, and this was in good agreement with past research that demonstrated that with an increase in the number of TiO_2 active sites, the photocatalytic efficiency was also increased [7,13,14]. Hence, the presence of TiO_2 coated 2D-PhC was directly responsible for the significant enhancement of the photocatalytic degradation of CR and AB-10B.

D. Supplementary Figures

S1. Molecular Structure of Model Dyes

Dye	Structure
Congo Red<br>$C_{32}H_{22}N_6Na_2O_6S_2$	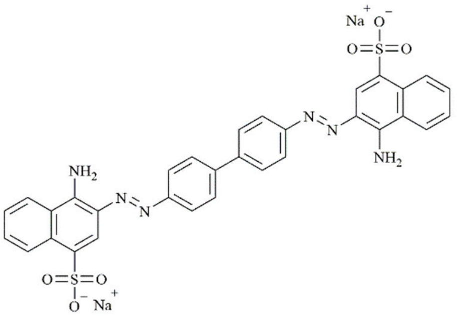
Amido Black 10B<br>$C_{32}H_{22}N_6Na_2O_6S_2$	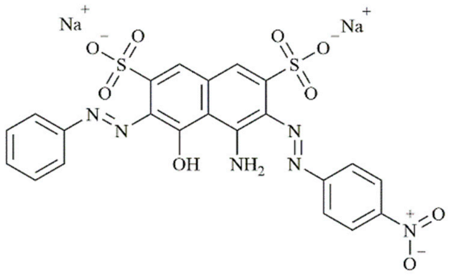

Figure S1. Molecular structures of Congo Red (CR) and Amido Black (AB-10B).

S2. Schematic of Experimental Setup for the Detection of AB-10B

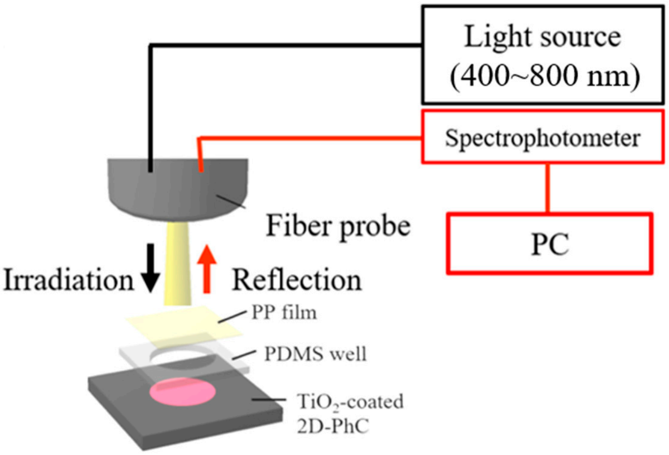

Figure S2. Schematic diagram of the reaction chamber and optical setup for AB-10B.

S3. Schematic of Experimental Setup for Detection of CR

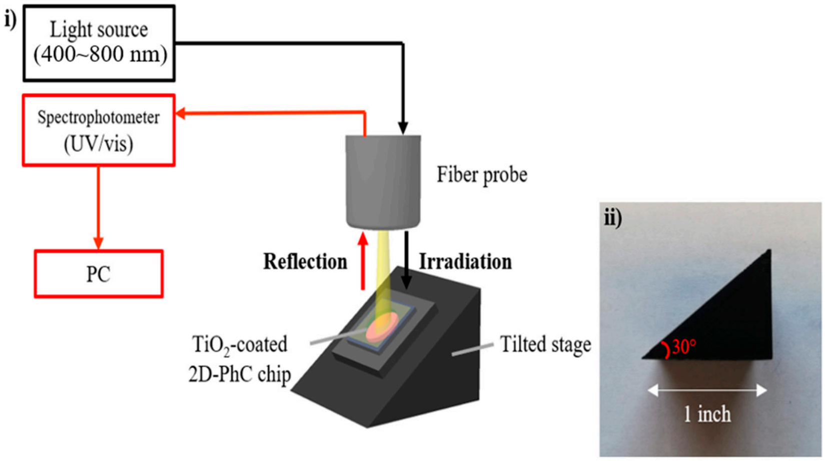

Figure S3. Schematic diagram of the optical setup used in the detection of CR. (i) The TiO_2 coated 2D-PhC placed on a tilted stage such that the incident angle of light irradiated on the TiO_2 surface was at 60° and (ii) the dimensions of the tilted stage used for obtaining the incident angle of 60°.

S4. Low Resolution XPS Survey Scans

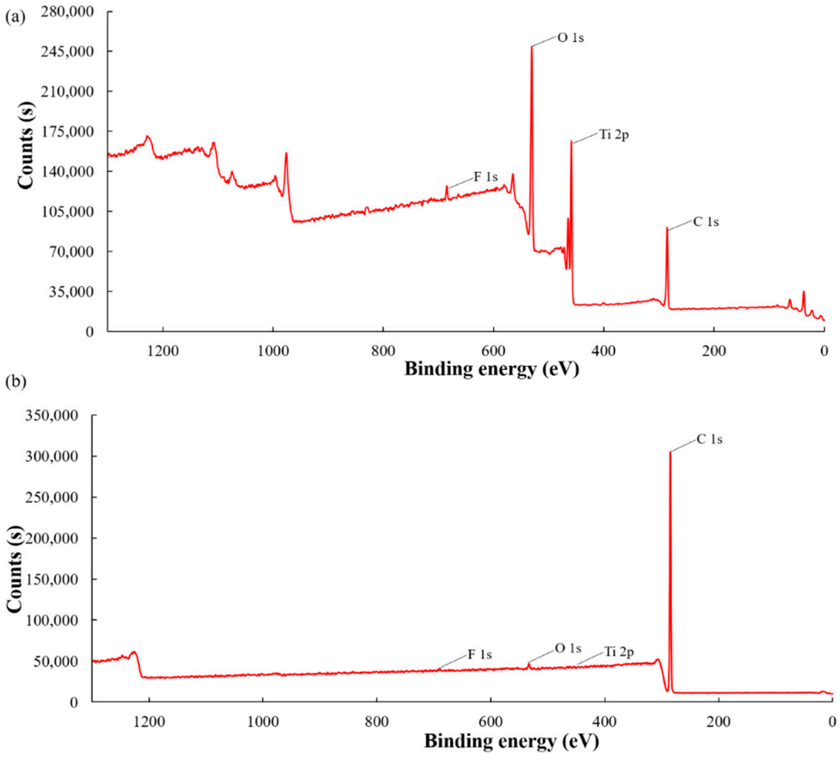

Figure S4. XPS survey scans for (**a**) 2D-PhC and (**b**) TiO_2 coated 2D-PhC surfaces.

S5. UV-Vis Absorbance Results Corresponding to the Color Change upon Different UV Irradiation Times

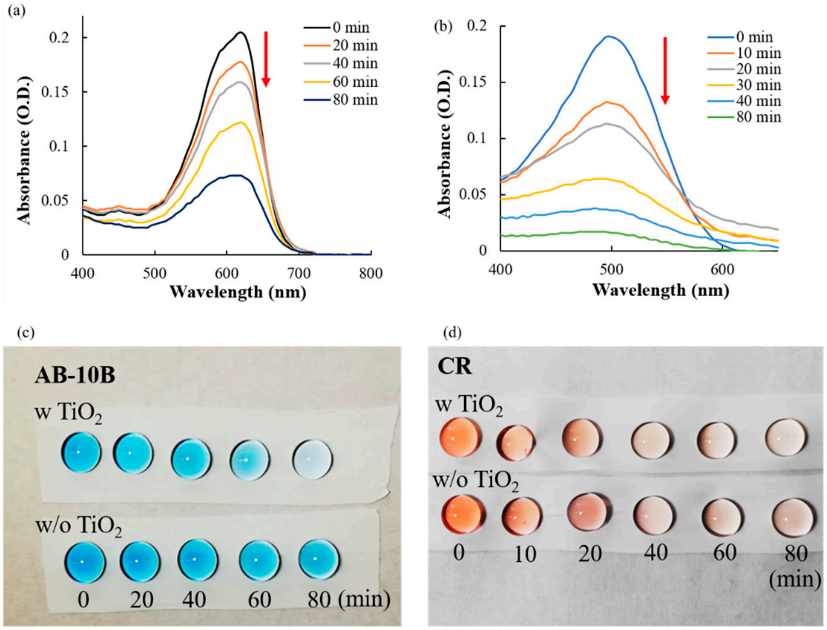

Figure S5. UV mediated degradation of diazo dyes using TiO_2 coated 2D-PhC. (**a**) UV-Vis absorption spectrum change representing time dependent degradation of 20 μM (**a**) AB-10B and (**b**) CR using TiO_2 coated 2D-PhC before and after UV treatment. Visual image of color change in 20 μM (**c**) AB-10B and (**d**) CR after UV treatment in the presence and absence of TiO_2 coated 2D-PhC at different time points.

The absorbance peak maxima for CR (~495 nm) and AB-10B (~620 nm) were monitored for the photocatalytic degradation after UV exposure at various time points. The degradation of the AB-10B (Figure S6a) and CR (Figure S6b) was confirmed by observing the peak absorbance intensity at respective wavelengths for peak maxima [15], which for both dyes decreased with increasing UV exposure time (Figure S6a,b). From Figure S6c,d, it was evident that the decolorization was more prominent and efficient when the dyes were UV irradiated on the TiO_2 coated 2D-PhC surface, suggesting the importance of TiO_2 in photocatalytic degradation of azo dyes, which was in agreement with past research [16,17]. The overall decrease in the absorption intensity (Figure S6a,b) signified the decrease in dye concentrations and complete photodegradation of the conjugate structure of the diazo dyes [15]. The decolorization kinetic rate (Figure S6c,d and Figure 6) of CR was faster than that of AB-10B, and it could be attributed to the higher non-specific adsorption yield of the CR compared to AB-10B due to its greater molecular weight [18,19].

S6. Reusability Tests for TiO_2 Coated 2D-PhC in Photocatalytic Degradation of CR and AB-10B

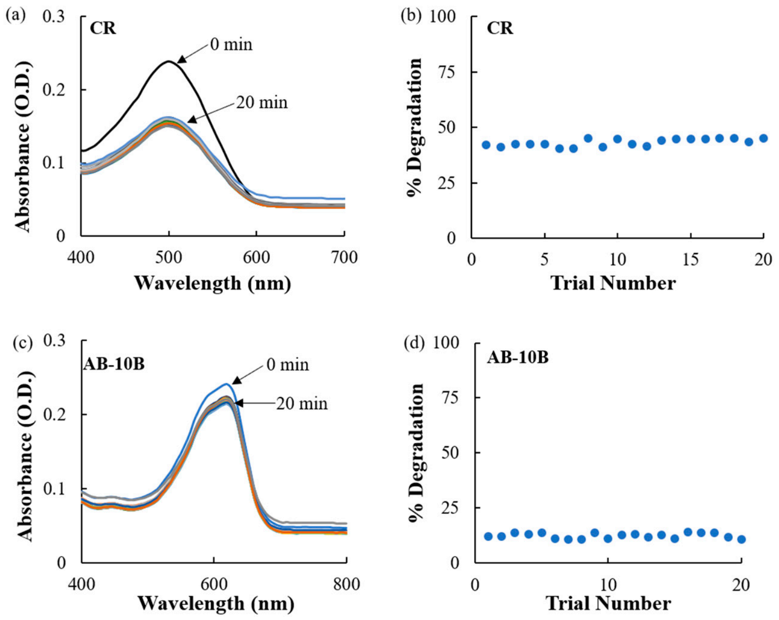

Figure S6. (**a**) An overlay of the absorbance spectra obtained before (0 min) and after 20 successive UV mediated photocatalytic degradation trials for 20 μM CR after 20 min. (**b**) The amount of photocatalytic degradation of CR after 20 min of UV irradiation across 20 successive trials. (**c**) An overlay of absorbance spectra obtained before (0 min) and after 20 successive UV mediated photocatalytic degradation trials for 20 μM AB-10B after 20 min. (**d**) The amount of photocatalytic degradation of AB-10B after 20 min of UV irradiation across 20 successive trials.

References

1. Endo, T.; Ozawa, S.; Okuda, N.; Yanagida, Y.; Tanaka, S.; Hatsuzawa, T. Reflectometric detection of influenza virus in human saliva using nanoimprint lithography-based flexible two-dimensional photonic crystal biosensor. *Sens. Actuat. B* **2010**, *148*, 269–276.
2. Aki, S.; Maeno, K.; Sueyoshi, K.; Hisamoto, H.; Endo, T. Development of a polymer/TiO_2 hybrid two-dimensional photonic crystal for highly sensitive fluorescence-based ion sensing applications. *Sens. Actuat. B* **2018**, *269*, 257–263.
3. Nishiguchi, K.; Sueyoshi, K.; Hisamoto, H.; Endo, T. Fabrication of gold-deposited plasmonic crystal based on nanoimprint lithography for label-free biosensing application. *Jpn. J. Appl. Phys.* **2016**, *55*, 08RE02.

4. Konstantinou, I.K.; Albanis, T.A. TiO_2 assisted photocatalytic degradation of azo dyes in aqueous solution: Kinetic and mechanistic investigations: A review. *Appl. Catal. B Environ.* **2004**, *49*, 1–14.
5. Akpan, U.G.; Hameed, B.H. Parameters affecting the photocatalytic degradation of dyes using TiO_2-based photocatalysts: A review. *J. Hazard. Mater.* **2009**, *170*, 520–529.
6. Chen, C.C.; Lu, C.S.; Chung, Y.C.; Jan, J.L. UV light induced photodegradation of malachite green on TiO_2 nanoparticles. *J. Hazard. Mater.* **2007**, *141*, 520–528.
7. Ma, Y.; Ni, M.; Li, S. Optimization of Malachite Green Removal from Water by TiO_2 Nanoparticles under UV Irradiation. *Nanomaterials* **2018**, *8*, 428.
8. Zhang, R.; Zeng, F.; Pang, F.; Ge, J. Substantial enhancement toward the photocatalytic activity of CdS quantum dots by photonic crystal-supporting films. *ACS Appl. Mater. Interfaces* **2018**, *10*, 42241–42248.
9. Li, P.; Wang, Y.; Wang, A.J.; Chen, S.L. TiO_2 activity enhancement through synergistic effect of photons localization of photonic crystals and the sensitization of CdS quantum dots. *Photonics Nanostructures - Fundam. Appl.* **2017**, *23*, 12–20.
10. Zheng, X.; Meng, S.; Chen, J.; Wang, J.; Xian, J.; Shao, Y.; Fu, X.; Li, D. Titanium dioxide photonic crystals with enhanced photocatalytic activity: Matching photonic band gaps of TiO_2 to the absorption peaks of dyes. *J. Phys. Chem. C* **2013**, *117*, 21263–21273.
11. Ma, Y.; Liu, H.; Han, Z.; Yang, L.; Liu, J. Non-ultraviolet photocatalytic kinetics of $NaYF_4$:Yb,Tm@TiO_2/Ag core@comby shell nanostructures. *J. Mater. Chem. A* **2015**, *3*, 14642–14650.
12. Lee, P.W.; Ong, S.T.; Hung, Y.T.; Lee, S.L. Photodegradation of malachite green by immobilization of titanium dioxide on glass plates. *Asian J. Chem.* **2013**, *25*, 755.
13. Bharati, B.; Sonkar, A.K.; Singh, N.; Dash, D.; Rath, C. Enhanced photocatalytic degradation of dyes under sunlight using biocompatible TiO_2 nanoparticles. *Mater. Res. Express* **2017**, *4*, 085503.
14. Mozia, S.; Morawski, A.W.; Toyoda, M.; Inagaki, M. Application of anatase-phase TiO_2 for decomposition of azo dye in a photocatalytic membrane reactor. *Desalination* **2009**, *241*, 97–105.
15. Modirshahla, N.; Behnajady, M.A. Photooxidative degradation of Malachite Green (MG) by UV/H_2O_2: Influence of operational parameters and kinetic modeling. *Dyes and Pigment* **2006**, *70*, 54–59.
16. Kashyap, J.; Ashraf, S.M.; Riaz, U. Highly efficient photocatalytic degradation of Amido Black 10B dye using polycarbazole-decorated TiO_2 nanohybrids. *ACS Omega* **2017**, *2*, 8354–8365.
17. Harun, N.S.; Abdul Rahman, M.N.; Wan Kamarudin, W.F.; Irwan, Z.; Muhammud, A.; Mohd. Akhir, N.E.F.; Yaafar, M.R. Photocatalytic degradation of congo red dye based on titanium dioxide using solar and UV lamp. *J. Fundam. Appl. Sci.* **2018**, *10*, 832–846.
18. Natarajan, S.; Bajaj, H.C.; Tayade, R.J. Recent advances based on the synergetic effect of adsorption for removal of dyes from waste water using photocatalytic process. *J. Environ. Sci.* **2018**, *65*, 201–222.
19. Costa, D.J.E.; Santos, J.C.S.; Sanches-Brandão, F.A.C.; Ribeiro, W.F.; Salazar-Banda, G.R.; Araujo, M.C.U. Boron-doped diamond electrode acting as a voltammetric sensor for the detection of methomyl pesticide. *J. Electroanal. Chem.* **2017**, *789*, 100–107.

© 2019 by the authors. Submitted for possible open access publication under the terms and conditions of the Creative Commons Attribution (CC BY) license (http://creativecommons.org/licenses/by/4.0/).
